# A Comparison of Prenatal Exposures in Children with and without a Diagnosis of Autism Spectrum Disorder

**DOI:** 10.7759/cureus.5223

**Published:** 2019-07-24

**Authors:** Alexandra Saunders, Jennifer Woodland, Sarah Gander

**Affiliations:** 1 Internal Medicine, Saint John Regional Hospital, Saint John, CAN; 2 Research Services, Saint John Regional Hospital, Saint John, CAN; 3 Pediatrics, Saint John Regional Hospital, Saint John, CAN

**Keywords:** autism, prenatal, environment, environment, environment, smoking, smoking, vitamins, vitamins

## Abstract

The current study was a case-control, focused on the presence of environmental exposures during pregnancy in mothers of children diagnosed with autism spectrum disorder (ASD) and children who were not. Exposures investigated included: acetaminophen/paracetamol use, air pollution, fever, smoking, parental age, maternal diabetes, prenatal vitamin use, workplace exposures, recreational drug use, seafood consumption, obesity, and maternal thyroid issues. Two-hundred and fifteen mothers of children (107 with ASD and 108 without ASD) aged 0-10 years participated in a telephone survey regarding prenatal exposures followed by a chart review. Data were analyzed with a series of univariate tests and a multivariate logistic regression. Univariate analyses showed correlation for the presence of siblings with ASD, presence of family members with ASD, maternal use of medications and maternal smoking during pregnancy; and child's gestational age at the start of prenatal vitamins with a diagnosis of ASD. Multivariate logistic regression analysis demonstrated an association with the use of medications (although specific medications could not be delineated due to small sample size), smoking, and gestational age at the start of prenatal vitamins. These preliminary results suggest that certain prenatal exposures (medication use, smoking, and gestational age at the start of prenatal vitamins) may be associated with a later diagnosis of ASD. Future research should be conducted with larger sample sizes and control for potentially confounding factors. Working towards an understanding of factors that come together to create or prevent a diagnosis of autism will be helpful for families, physicians, and allocating government resources.

## Introduction

Autism spectrum disorder (ASD) is a multifactorial disorder characterized by varying deficits in social interactions, disordered communication, and repetitive behaviour patterns. Signs that a child has autism are present in the early developmental stages and the symptoms cause significant impairment in many areas of functioning, including social, educational/occupational, and performance of everyday activities. According to the Diagnostic and Statistical Manual of Mental Disorders - Fifth Edition (DSM-5), ASD can be divided into three levels of severity: requiring very substantial support, requiring substantial support, and requiring support [1]. One of several specifiers can follow a diagnosis including presence or absence of intellectual impairment, language impairment, a known medical condition, another neurodevelopmental, mental or behavioural disorder, or catatonia [2].

There have been established genetic correlates and heredity with ASD diagnoses; however, chromosomal changes have only been found in approximately 25% of children studied with no single variance predominating [3]. This signifies that there may be other external factors at play for autism to develop from genetic risk. Studies suggest that prenatal rather than postnatal exposures are significant due to disruption of neuron gene networks in the cell cycle, protein folding, DNA damage repair and cell apoptosis [4].

Potential prenatal exposures are the focus of this study, with respect to one geographical area. Many environmental pathogens are known to affect neurological function; a non-exhaustive list provided in a review by Diav-Citrin includes “infectious agents such as rubella, cytomegalovirus (CMV), or Toxoplasma; drugs (e.g., antiepileptic drugs, valproate, for instance, or retinoids); Substances of abuse like alcohol; Chemicals, lead for example; and Physical factors like ionizing radiation” [5]. The review recognized that exposures can lead to congenital malformations creating neural tube defects, disorders in cell organization, and/or problems with metabolism. Many substances were linked to a higher incidence of autism spectrum disorder. Maternal metabolic conditions have also been related to neurodevelopmental problems [6]. Furthermore, there also is evidence to suggest that the geographic location of the mother during pregnancy may also play a role in the diagnosis of ASD [7].

The purpose of the study was to investigate a combination of previously discussed external factors, including acetaminophen/paracetamol use, air pollution, fever, parental age, maternal diabetes, prenatal vitamin use, workplace exposures, recreational drug use, seafood consumption, maternal thyroid issues, and obesity [5, 8-15].

## Materials and methods

Participants were recruited from a medium-sized city in Atlantic Canada, consisting of children aged 0-10, diagnosed with ASD before age six. Children with another developmental diagnosis or chromosomal abnormality, children of adoptive parents, and children whose caregiver is a single male were excluded from the study. A total of 181 children were identified via patient charts, databases of four local pediatricians, and those who self-identified after seeing recruitment posters. The total number of females was 49 and males were 132. From these groups, 39 females and 102 males were selected randomly by use of a random number table.

The comparison group (‘non-ASD’) was a random sample of children from the same region of the city and within the same age range as the ASD group selected after they contacted the Research team after seeing study recruitment posters. Females and males were selected separately, to maintain the gender ratio as above. 55 females and 144 males were randomly selected. Finally, the current study was approved by a local Research Ethics Board and the mothers of all participants gave informed consent prior to participating and were told the study was gathering information on prenatal factors related to ASD. Prenatal exposure information was gathered from each participant's mother (see Table 1). 

**Table 1:** Telephone Survey Questions ASD: Autism spectrum disorder

Background Information			
Question Topic	Yes	No	Follow-Up Question
Gestational age at discovery of pregnancy			Weeks: Months:
Other children diagnosed with ASD	Yes	No	
Family members diagnosed with ASD	Yes	No	Who:
Age of father at beginning of pregnancy			Father’s Age:
Exposure Questions			
Question Topic	Yes	No	Follow-Up Question to Yes
Fever	Yes	No	# Days of exposure
Acetaminophen use	Yes	No	# Doses
Antibiotic use	Yes	No	Which antibiotics?
Medication use (all categories other than acetaminophen and antibiotics)	Yes	No	Which medications?
Prenatal vitamin use	Yes	No	Gestational age at start of prenatal use
Cigarette use	Yes	No	Number smoked per day
Marijuana use	Yes	No	Amount smoked per day
Alcohol use	Yes	No	-Prior to pregnancy discovery? -Number of drinks
Recreational drug use	Yes	No	Drugs used
Seafood consumption	Yes	No	Meals of seafood total
Living in area with noticeable air pollution	Yes	No	Location within city area
Work during pregnancy	Yes	No	Location of work
Exposures in the workplace	Yes	No	Exposure type

In the ASD group, the total number of mothers, with female and male children, was 28 and 79 respectively, giving a total of 107 participants. For the comparison group, the total number of mothers who participated was 33 (female child) and 75 (male child), for a total of 108.

The mothers of these participants were contacted by mail and/or telephone and were asked to take part in a telephone survey which took approximately 10-15 minutes to complete (see Table 1). Following the telephone survey, a chart review of the maternal-prenatal record and the medical record of the child was performed. The prenatal record was used to collect information regarding the mother’s age at the start of pregnancy, height, weight, oral glucose tolerance test result, presence of gestational diabetes, past pregnancies, miscarriages/stillbirths, and presence of thyroid condition. The child’s medical record was used to gain information regarding their gestational age at birth, age at autism diagnosis (for those in ASD group), and review for other developmental challenges included in the exclusion criteria.

All data were analyzed using IBM SPSS Statistics for Windows, Version 22. Due to the many variables investigated, the analysis was done in two stages. First, a series of chi-square or independent t-tests were performed on variables as appropriate. Second, variables that were statistically significant in the first stage were entered into a multivariate logistic regression model, along with one variable that approached significance. Due to gaps in prenatal records, 14/213 cases could not be analyzed as portions of the data were missing and were therefore excluded from the analysis.

## Results

At the time of their pregnancy mothers had an average age of 27.98 years (SD= 6.12), an average height of 164 cm (SD= 6.39), an average weight of 83.9 kg (SD= 21.09). Fathers had an average age of 30.8 years (SD= 7.11). Children ages ranged from five months to 10 years, and of the children who had autism, the average age at diagnosis was 3.2 years (SD= 1.36). There was no statistically significant difference between the mean age of mothers of children diagnosed with ASD (27.54 years (SD= 6.26)) and mothers of children not diagnosed with ASD (28.14 years (SD= 6.72), t(198)= -0.65, p= 0.515) (Table 2). Thus, subsequent analyses were not adjusted for age.

**Table 2:** Independent t-test results by exposure variable EGA: estimated gestational age ASD: autism spectrum disorder

Variable	Means	Standard Deviation	t	Degrees of Freedom	Significance (p)
Age of father	ASD: 30.0388	7.13445	-1.505	209	0.134
	Non-ASD: 31.5093	7.05717			
Mother’s height	ASD: 164.0619	6.04982	0.231	196	0.818
	Non-ASD: 163.8515	6.73259			
Mother’s weight	ASD: 85.8542	22.51081	1.271	193	0.205
	Non-ASD: 82.0202	19.55838			
Oral glucose tolerance test	ASD: 6.3394	1.49150	0.992	189	0.323
	Non-ASD: 6.1330	1.38366			
Mother’s age	ASD: 27.5361	6.26342	-0.652	198	0.515
	Non-ASD: 28.1359	6.71704			
Child’s EGA at birth	ASD: 38.4118	2.72267	1.077	199	0.283
	Non-ASD: 37.9596	3.21641			
Days with fever	ASD: 3.7000	3.46562	-0.617	29	0.542
	Non-ASD: 4.8182	6.69056			
EGA at start of prenatal vitamins	ASD: 5.6633	4.35995	2.981	199	0.003*
	Non-ASD: 3.9417	3.82157			
Number of past pregnancies	ASD: 1.4742	1.83197	0.652	197	0.515
	Non-ASD: 1.3235	1.40833			
Number of miscarriages	ASD: 0.6495	1.34661	0.682	197	0.496
	Non-ASD: 0.5294	1.13195			
Number of seafood meals	ASD: 15.3524	34.05340	1.528	211	0.128
	Non-ASD: 9.8056	15.99384			

Some variables were excluded from the regression analysis as they violated the cell count assumption of the Chi-square test. A multivariate logistic regression analysis was performed. The outcome variable was the diagnosis of ASD. Based on statistically significant Chi-square and t-tests, the variables in the regression model included the presence of siblings with ASD, presence of family members with ASD, presence of fever, use of medications (other than acetaminophen or antibiotics), smoking, and gestational age at the start of prenatal vitamins (Tables 2-4). Note that although fever was not statistically significant (p=0.067), it approached significance and was included (Table 3).

**Table 3:** Chi-square test significance by exposure variable ASD: autism spectrum disorder

	Pearson Chi-Square	
Variable	χ^2^	p
Sibling with ASD	13.189	0.000*
Family member with ASD	14.890	0.000*
Presence of fever	3.363	0.067
Acetaminophen use	1.612	0.657
Antibiotic use	0.550	0.458
Medication use	9.557	0.002*
Prenatal use	0.416	0.519
Cigarette use	19.679	0.000*
Alcohol use	0.083	0.773
Seafood consumption	2.086	0.149
Gestational diabetes present	2.392	0.122
Thyroid condition present	1.245	0.264

**Table 4:** Chi-square test group numbers and total percentages by exposure variable ASD: autism spectrum disorder

	ASD		Non-ASD	
	N	%	N	%
ASD siblings				
Yes	33	15.5	12	5.6
No	72	33.8	96	45.1
ASD Family				
Yes	40	18.8	16	7.5
No	65	30.5	92	43.2
Fever				
Yes	20	9.4	11	5.2
No	85	39.9	97	45.5
Medications				
Yes	60	28.4	39	18.5
No	44	20.9	68	32.2
Smoking				
Yes	43	20.2	15	7.0
No	62	29.1	93	43.7

The logistic regression model was statistically significant, X2 (6) = 42.67, p < .001, and explained 25.7% (Nagelkerke R2) of variance in ASD diagnosis. Variables found to be statistically significant included presence of family members with ASD, χ2 (1, N=199) = 6.91, p < 0.05, use of medications, χ2 (1, N=199) = 6.53, p < 0.05, use of cigarettes, χ2 (1, N=199) = 5.80, p < 0.05, and gestational age at the start of prenatal vitamins, χ2 (1, N=199) = 4.09, p < 0.05 (Table 5). Thus, the odds of an autism diagnosis increased if there was a family member with autism (Odds Ratio (OR) = 2.72; 95% CI [1.29, 5.73]) as opposed to not having a family member with autism, if the mother used medications (other than acetaminophen or antibiotics) during the pregnancy (OR = 2.29; 95% CI [ 1.21, 4.34]) as opposed to not using medications, if the mother smoked cigarettes during the pregnancy (OR = 2.56; 95% CI [1.19, 5.49]) as opposed to not smoking cigarettes. In addition, of those who took prenatal vitamins during pregnancy, increasing gestational age at the start of prenatal vitamins was associated with an increased likelihood of ASD diagnosis (OR = 1.08; 95% CI [1.00, 1.18]) (Table 5). 

**Table 5:** Logistic regression statistics Odds ratios and 95% confidence intervals for factors included in logistic regression

Variable	p	Odds ratio	95% Confidence Interval	
			Lower	Upper
ASD Siblings	0.109	2.06	0.85	4.96
ASD Family	0.009	2.72	1.29	5.73
Fever	0.19	1.87	0.73	5.73
Medications	0.011	2.29	1.21	4.36
Smoking	0.016	2.56	1.91	5.49
Gestational age at start of prenatal vitamins	0.043	1.08	1.00	1.8

## Discussion

This study provides preliminary evidence to suggest that prenatal exposures such as the use of medications (other than acetaminophen or antibiotics, some examples including antihypertensives, antidepressants, benzodiazepines, and steroids), use of cigarettes, gestational age at the start of prenatal vitamins, and whether a family member was diagnosed with ASD may be associated with the child being diagnosed with ASD. With a large number of variables and smaller sample sizes per group, each of these variables was only significant on the feature of presence or absence of said variable. The number of cigarettes smoked, types of medications used, and the formulation of the prenatal vitamin could not be assessed statistically.

**Figure 1:**
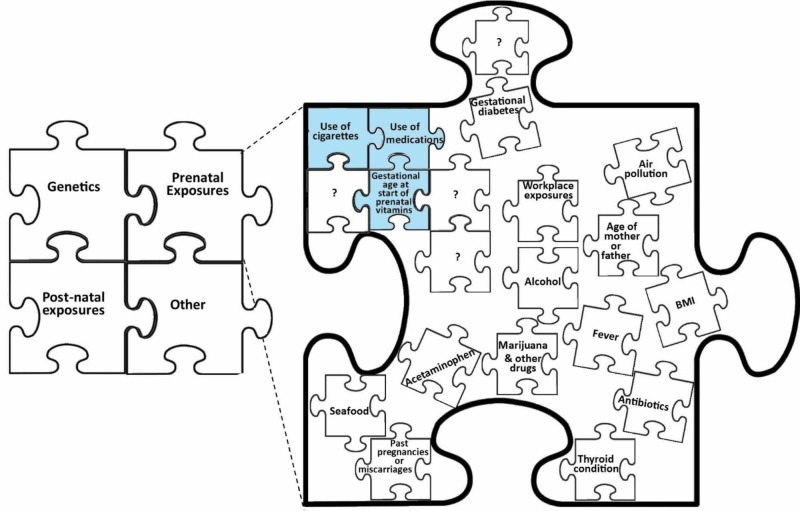
A visualization of the "pieces of the puzzle" that combine to create autism spectrum disorder, with a narrowed-in view of prenatal exposure The pieces shaded-in and connected were found to be significant in our study, and the free-floating pieces were not found to be significant. Many factors (?) remain unknown and will require further studies.

The variables in the regression may correlate with a later diagnosis of ASD, therefore more information (e.g., socioeconomic status (SES)) is necessary. Research has suggested that SES is associated with ASD diagnoses [3-4]. Additionally, there is evidence suggesting that individuals of lower SES are more likely to use cigarettes [5]. Therefore, the use of cigarettes may no longer be associated with ASD diagnosis after adjusting for SES. Without controlling for these missing pieces, the current model cannot fully account for all variables in the complex diagnosis of ASD. Future research could build on the results of the study by adjusting for confounding variables. This small study sheds light on a few pieces of the puzzle that is ASD, and there are still more pieces to be found (Figure 1).

Another potential limitation of the present study is related to questions regarding alcohol consumption. The results from the questionnaire were self-reported and no participants admitted to drinking after the discovery of their pregnancy (within the first-month gestational age). While this may be a true finding, as other participants did reveal their illicit drug use, it is possible that mothers were not entirely honest with answering this specific question. The nature of the survey (i.e., by telephone) may have caused them to feel self-conscious. In addition, the survey relied on recall at the time of pregnancy potentially causing reduced accuracy. In future studies, the age of the children could be reduced to decrease the time since pregnancy at the time of interviewing.

Interestingly, seafood consumption was higher in the non-ASD group, with 53.7% compared to 43.8% of the ASD group, albeit differences were not statistically significant. Reasons for this may be a poor recall or biased recall in mothers of children with ASD; mercury content of seafood and the association of mercury with ASD is popular public knowledge. Correcting for SES would also play a role in the purchase of seafood/fish.

With regards to location, 27% of those with ASD lived in outlying regions of the city, compared to 36% of the non-ASD group. This factor was investigated to see if participants living in an area with higher amounts of pollution experienced more diagnoses of ASD. While this data was unable to be interpreted statistically due to low responses for some regions, it leaves an interesting area for future study. The inner-city region the area in question has two large industries (oil refinery and paper mill), and this pollution may play a role in ASD development, a relationship shown in previous studies [16]. Location may play a role with regards to areas of high and low socioeconomic status (SES). One example in our data set is from the “North” region, which has a historically higher rate of poverty: 21% of those in the ASD group lived North, compared to 9% of non-ASD [9]. Future studies with measured levels of pollution and SES will be needed to determine the true importance of these findings.

Another interesting feature of the data was the ratio of females to males. In previous studies, the ratio was 1:4 - 1:5 (females to males). The current sample had a ratio of 1:2.7. There are many possible reasons for this difference. One is that this group is not representative of the whole region. Future research, with more participants, would help to determine if this is the case. Another reason could be the gene pool in the area of study. Previous studies hypothesized that females have a higher genetic threshold and will, therefore, develop ASD at a lower rate compared to males with similar exposures [15]. Lastly, with increased vigilance and detection of ASD, the gender gap may be smaller than in previous studies.

An additional area of study for the future would be to determine if there is a higher rate of autism in our city in comparison to the rest of the province. If so, features specific to this location could be isolated used to aid in reduction and prevention strategies in cities across the country; understanding autism predictors would be of great value to pediatricians in any city. 

## Conclusions

Autism spectrum disorder remains a multifactorial developmental condition with many causational factors unknown. The goal of this study was to shed light on environmental factors associated with ASD diagnosis, though the nature of a retrospective study does not allow for suggestions of causation. The use of medications, use of cigarettes, and later gestational age at the start of prenatal vitamins were correlated with ASD diagnosis. Areas of direction for further research include replicating this study with an increased sample, reducing the number of variables, controlling for SES, and other potentially confounding variables. In addition, epidemiological studies comparing rates of ASD diagnoses to other regions and provinces would provide useful information. Understanding external exposures associated with ASD would have a large impact on family functioning and could potentially save the Canadian economy money and resources. From a medical practice perspective, primary care physicians and pediatricians could help to advocate for change of unavoidable exposures (like pollution) and to give advice regarding factors that can be avoided.
